# CBP/p300 is a cell type-specific modulator of CLOCK/BMAL1-mediated transcription

**DOI:** 10.1186/1756-6606-2-34

**Published:** 2009-11-19

**Authors:** Hiroshi Hosoda, Kenichi kato, Hidenori Asano, Motonori Ito, Haruno Kato, Taku Iwamoto, Akinobu Suzuki, Shoichi Masushige, Satoshi Kida

**Affiliations:** 1 Department of Bioscience, Faculty of Applied Bioscience, Tokyo University of Agriculture, 1-1-1 Sakuragaoka, Setagaya-ku, Tokyo 156-8502, Japan; 2 Department of Agricultural Chemistry, Faculty of Applied Bioscience, Tokyo University of Agriculture, 1-1-1 Sakuragaoka, Setagaya-ku, Tokyo 156-8502, Japan

## Abstract

**Background:**

Previous studies have demonstrated tissue-specific regulation of the rhythm of circadian transcription, suggesting that transcription factor complex CLOCK/BMAL1, essential for maintaining circadian rhythm, regulates transcription in a tissue-specific manner. To further elucidate the mechanism of the cell type-specific regulation of transcription by CLOCK/BMAL1 at the molecular level, we investigated roles of CBP/p300 and tissue-specific cofactors in CLOCK/BMAL1-mediated transcription.

**Results:**

As shown previously, CBP/p300 stimulates CLOCK/BMAL1-mediated transcription in COS-1 cells. However, CBP/p300 repressed CLOCK/BMAL1-mediated transcription in NIH3T3 cells and knockdown of CBP or p300 expression by siRNA enhanced this transcription. Studies using GAL4-fusion proteins suggested that CBP represses CLOCK/BMAL1-mediated transcription by targeting CLOCK. We further investigated mechanisms of this cell type-specific modulation of CLOCK/BMAL1-mediated transcription by CBP by examining roles of co-repressor HDAC3 and co-activator pCAF, which are highly expressed in NIH3T3 and COS cells, respectively. CBP repressed CLOCK/BMAL1-mediated transcription in COS-1 cells when HDAC3 was overexpressed, but activated it in NIH3T3 cells when pCAF was overexpressed. CBP forms a complex with CLOCK by interacting with HDAC3 or pCAF; however, direct interaction of CBP with CLOCK was not observed.

**Conclusion:**

Our findings indicate possible mechanisms by which CBP/p300 tissue-specifically acts cooperatively with pCAF and HDAC3 either as a co-activator or co-repressor, respectively, for CLOCK/BMAL1.

## Background

Physiological and behavioral systems in many organisms manifest a circadian rhythm that is regulated by an endogenous clock with a period of approximately 24 hrs [1,2]. Eukaryotic cells, including immortalized cells in culture, retain circadian rhythms and circadian oscillation in patterns of cellular transcription [3-6]. In mammals, the SCN of the anterior hypothalamus contain neurons that function as a master clock generating the circadian rhythm [7-9]. The SCN is thought to synchronize the timing of circadian oscillation in cells throughout the body.

Recent molecular and genetic studies in flies and rodents have identified a set of clock genes, the loss of function of which leads to an impairment of the normal circadian rhythm [1,2,10,11]. In particular, a genetic screen in mice revealed that the bHLH-PAS transcription factor, CLOCK, is a master clock gene [12]. Subsequently, BMAL1 was shown to form a heterodimer with CLOCK [13,14]. The CLOCK/BMAL1 complex has been shown to bind a specific DNA sequence (the E-box) [13,14] and to activate transcription of core clock genes, including *period *1, 2 and 3 and *cryptochrome *1 and 2, which in turn inhibit CLOCK/BMAL1 activity [2,13,15-18]. In addition, PERIOD 2 suppresses the activity of Rev-Erb, which also inhibits CLOCK/BMAL1 function [2,19,20]. These positive and negative feedback loops are central to the generation of cellular circadian oscillation.

The CLOCK/BMAL1 heterodimer regulates circadian transcriptional oscillations of core clock genes in both the SCN and peripheral tissues [1,2,11,15,16,18,21]. Comparison of the transcriptional rhythm of CLOCK/BMAL1 target genes in different tissues indicates that although the rhythmic expression of *period *mRNA is similar in all, there are minor differences in the waveform and amplitude of the cycle of expression, even when the phases of peak expression are normalized [6,22,23]. Additionally, although these CLOCK/BMAL1-regulated genes are expressed in multiple tissues, their levels of expression differ both in peripheral tissues and in the brain [6,18,24-26]. These observations raise the possibility that CLOCK/BMAL1 activity is modified not only by PERIODs and CRYs, but also by other tissue-specific transcriptional regulators.

The activation of transcription in eukaryotic cells is mediated by both activators that bind to specific DNA sequences and co-activators that do not directly bind DNA, but instead form multiprotein complexes with other initiation factors, other cofactors and other activators [27,28]. Co-activators facilitate the initiation of transcription through either an interaction with both activators and the initiation complex, or through a role in remodelling or modifying chromatin structure [28,29]. CBP and its homologue p300 have been shown to function as co-activators for a number of transcriptional activators through direct interaction with these factors and/or through intrinsic HAT activity, which allows them to play a role in chromatin remodelling [30-33]. Interestingly, recent studies have shown that CBP/p300 complexes can act as transcriptional corepressors as well as coactivators [34,35].

In several cell lines (HEK293 cells, COS-7 cells and Hep3B cells), CBP and p300 have been shown to stimulate CLOCK/BMAL1 activity [36,37]. Consistent with this, both CBP and p300 form a complex with BMAL1, but not CLOCK, in HEK293 and Hep3B cells [36], strongly suggesting that CBP/p300 targets BMAL1. However, recent studies have demonstrated that p300, but not CBP, forms a complex with CLOCK and BMAL1 in liver extract [37], suggesting that only p300 acts as a coactivator for CLOCK/BMAL1. These contrasting observations suggest that the modification of CLOCK/BMAL1 activity by CBP/p300 may be tissue- or cell type-specific.

Our interest lies in understanding the mechanism of the cell type-specific regulation of transcription by CLOCK/BMAL1 at the molecular level. Here, we investigated the cell type-specific roles of CBP/p300 in CLOCK/BMAL1-mediated transcription. Surprisingly, although CBP enhanced CLOCK/BMAL1-mediated transcription in COS-1 cells, overexpression and RNAi-mediated knockdown experiments indicated that CBP and p300 repressed CLOCK/BMAL1-mediated transcription in NIH3T3 cells. More importantly, such positive and negative regulation of CLOCK/BMAL1-mediated transcription by CBP/p300 was mediated by the cell type-specific transcriptional co-activator pCAF and co-repressor HDAC3, respectively. These findings indicate possible mechanisms for cell type-specific modulation of CLOCK/BMAL1 activity by CBP/p300 and tissue-specific cofactors.

## Results

### CBP inhibits CLOCK/BMAL1-mediated transcription in NIH3T3 cells

**Figure 1:**
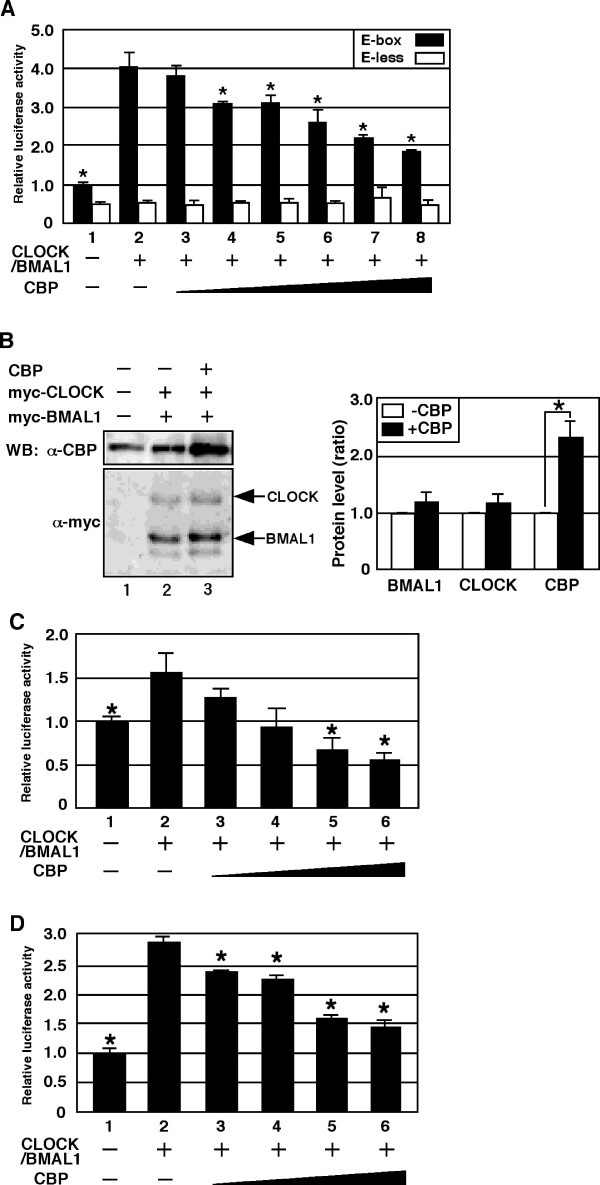
**CBP represses CLOCK/BMAL1-mediated transcription in NIH3T3 cells**. (A) Cells were transiently transfected with either pE-box (black-bar) or pE-less (white-bar) (2 ng), either with or without pcDNA3CLOCK (100 ng) and pcDNA3BMAL1 (100 ng), in the presence of increasing amounts (0, 5, 10, 20, 50, 100 or 200 ng) of pcDNA3CBP, as indicated. Empty vector (pcDNA3) was used to standardize the total amount of transfected DNA (502 ng). An asterisk indicates p < 0.05 compared with black-bar in lane 2 (pE-box; Student's *t *test). (B) Cells grown in six-well plates were transiently transfected with either pCMV-myc (1000 ng) or pmyc-CLOCK (500 ng) and pmyc-BMAL1 (500 ng), either alone or in combination with pcDNA3CBP (500 ng). Empty vector (pCMV-myc and pcDNA3) was used to standardize the total amount of transfected DNA (2 μg). 48 hrs later, transfected cells were lysed and cell lysates were subjected to Western blot analysis using anti-myc or anti-CBP antibody. Relative proteins level was presented. An asterisk indicates a *P *value of < 0.05 (Student's *t *test, n = 3). (C) pVasopressin-Luc or (D) pPeriod1-Luc (10 ng) were transiently cotransfected, with or without pcDNA3CLOCK (100 ng) plus pcDNA3BMAL1 (100 ng), in combination with increasing amounts (0, 20, 50, 100 or 200 ng) of pcDNA3CBP, as indicated. Empty vector (pcDNA3) was used to standardize the total amount of transfected DNA (510 ng). An asterisk indicates p < 0.05 compared with lane 2 (Student's *t *test).

Previous reports have indicated that CLOCK/BMAL1 activates E-box-mediated transcription [13,15,16]. To investigate the role of CBP in the activation of transcription by CLOCK/BMAL1, we examined the effects of forced expression of CBP in NIH3T3 cells. Consistent with previous reports, coexpression of CLOCK/BMAL1 activated transcription from a synthetic E-box-dependent promoter containing three copies of the E-box sequence from the *vasopressin *gene (pE-box; Figure 1A). However, forced coexpression of CBP along with CLOCK/BMAL1 inhibited activation of transcription by CLOCK/BMAL1 in a dose-dependent manner. In contrast, CBP did not affect the transcription of a promoter that did not contain an E-box. Overexpression of CBP did not affect the level of expression of either CLOCK or BMAL1 (Figure 1B). These results suggest that CBP inhibits CLOCK/BMAL1-mediated transcription specifically in NIH3T3 cells.

It is possible that the inhibitory effect of CBP on CLOCK/BMAL1-mediated transcription (Figure 1A) is specific to the synthetic E-box-dependent promoter used in this study. Therefore, we next examined whether CBP represses CLOCK/BMAL1-mediated transcription from natural promoters containing an E-box. Coexpression of CLOCK/BMAL1 led to activation of transcription from both promoters (Figure 1C, D). Consistent with the result in Figure 1A, coexpression of CBP along with CLOCK/BMAL1 also led to dose-dependent inhibition of CLOCK/BMAL1-mediated transcription (Figure 1C, D). Taken together, we conclude that CBP inhibits CLOCK/BMAL1-mediated transcription in NIH3T3 cells. This observation is not concordant with previous results indicating that CBP/p300 functions as a coactivator for CLOCK/BMAL1 in COS-7 and HEK 293 cells [36,37].

### CBP represses CLOCK/BMAL1-mediated transcription by targeting CLOCK in NIH3T3 cells

**Figure 2:**
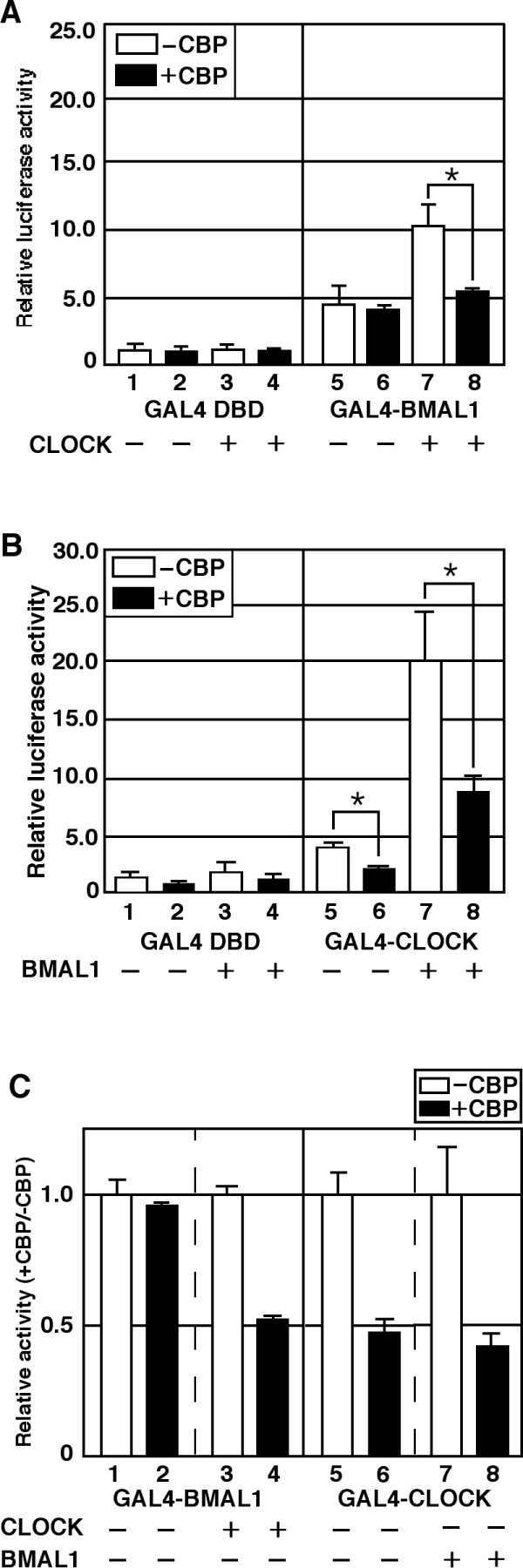
**CBP inhibits CLOCK/BMAL1 activity by targeting CLOCK in NIH3T3 cells**. (A) Cells were transiently transfected with pGAL4RE-Luc (5 ng), together with either pGAL4 or pGAL4-BMAL1 (100 ng), alone or in combination with pcDNA3CBP (100 ng) alone, pcDNA3CLOCK (100 ng) alone, or pcDNA3CBP and pcDNA3CLOCK, as indicated. (B) Cells were transiently transfected with pGAL4RE-Luc (5 ng), together with either pGAL4 or pGAL4-CLOCK (100 ng) alone or in combination with pcDNA3CBP (100 ng) alone, pcDNA3BAML1 (100 ng) alone, or pcDNA3CBP and pcDNA3BMAL1, as indicated. (C) Summary of Fig. 2 (A) and (B). The ratios of the transcriptional activity of each group with and without co-expression of CBP are indicated. An asterisk indicates p < 0.05 (Student's *t *test). Empty vector (pcDNA3) was used to standardize the total amount of transfected DNA (405 ng).

We next studied the mechanism by which CBP represses CLOCK/BMAL1-mediated transcription. To do this, we created fusions of CLOCK and BMAL1 with the GAL4-DNA binding domain. Both GAL4-BMAL1 and GAL4-CLOCK activated transcription from a promoter bearing GAL4 binding sites upstream of a TATA box (Figure 2A, B), indicating that both BMAL1 and CLOCK contain functional domains that can activate transcription. Coexpression of CLOCK or BMAL1 with GAL4-BMAL1 or GAL4-CLOCK, respectively, further stimulated transcription from this promoter (Figure 2A, B). It is important to note that we observed these interactions between CLOCK and BMAL1 under our experimental conditions using co-immunoprecipitation assays (see Additional file 1). These results indicate that heterodimerization of CLOCK and BMAL1 further stimulates transcription of promoters to which either is bound.

Coexpression of CBP along with GAL4-CLOCK or GAL4-BMAL1 inhibited transcription from this promoter mediated by GAL4-CLOCK, but not by GAL4-BMAL1 (Figure 2A, B). However, consistent with our previous observation using a synthetic E-box-dependent promoter, CBP repressed the activity of both GAL4-BMAL1 and GAL4-CLOCK when the partner of the heterodimer was coexpressed (Figure 2A, B). The inhibitory effects of CBP on the activities of GAL4-CLOCK, GAL4-CLOCK/BMAL1 and GAL4-BMAL1/CLOCK were comparable (Figure 2C). Therefore, these results indicate that CBP represses CLOCK/BMAL1-mediated transcription through an effect upon the CLOCK protein.

### Both the KIX and NR domains of CBP are required to inhibit CLOCK/BMAL1 activity

**Figure 3:**
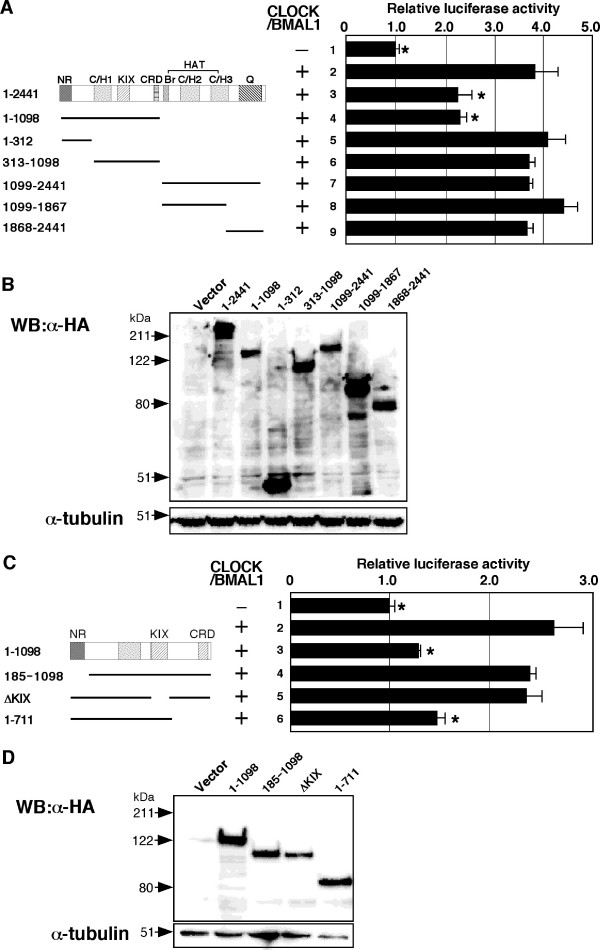
**The NR and KIX domains of CBP are required for the repression of CLOCK/BMAL1 activity**. (A), (C) NIH3T3 cells were transiently transfected with pE-box (2 ng), together with or without pcDNA3CLOCK (100 ng) plus pcDNA3BMAL1 (100 ng) in combination with pcDNA3CBP (100 ng) or pCMV-HA carrying the indicated CBP deletion mutants (1-1098, 1-312, 313-1098, 1099-2441, 1099-1867, 1868-2441, 185-1098, Δ KIX, 1-711; 100 ng), as indicated. Empty vector (pcDNA3) was used to standardize the total amount of transfected DNA (402 ng). An asterisk indicates p < 0.05 compared with lane 2 (Student's *t *test). (B), (D) Protein expression of wild-type CBP and CBP mutants was verified by Western blotting of NIH3T3 cell lysates using HA-antibody. α-tubulin serves as an equal loading control.

CBP contains multiple domains that contribute to the regulation of transcription by this factor (for example, the nuclear receptor interaction [NR] domain, the CREB binding domain [KIX], the cysteine-histidine rich [C/H] domain, the histone acetyltransferase [HAT] domain, the cell cycle regulatory domain 1 [CRD1], etc) [33,35]. Previous studies have indicated that the HAT domain is required for both the activation and repression of transcription ([33,34,38]) and that the CRD1 domain acts as a strong repressor of transcription [35]. We next investigated the region of CBP required for the inhibition of CLOCK/BMAL1-mediated transcription. CBP1-1098 inhibited CLOCK/BMAL1 activity as efficiently as did full-length CBP (Figure 3A). In contrast, mutants truncated at the N-terminus (CBP1098-2441, CBP1098-1867 or CBP1868-2441) did not inhibit CLOCK/BMAL1 activity (Figure 3A). These data indicate that the N-terminal region of CBP mediates the inhibition of CLOCK/BMAL1 activity.

However, CBP1-312 and CBP313-1098 failed to inhibit CLOCK/BMAL1-mediated transcription (Figure 3A). Because these regions contained the NR domain, and the KIX and CRD1 domains, respectively, we generated mutants only lacking each of these domains, and examined the effects of these mutations on the inhibition of CLOCK/BMAL1 activity. Interestingly, both CBP185-1098 (lacking the NR domain) and CBPΔKIX (lacking the KIX domain, residues 452-714) failed to repress CLOCK/BMAL1 activity, while CBP1-711 (lacking CRD1) was as efficient as CBP1-1098 in inhibiting CLOCK/BMAL1 (Figure 3C). It is important to note that comparable expression of wild-type CBP and CBP mutants were observed by Western blotting using anti-HA antibody (Figure 3B, D). These results indicate that both the NR and KIX domains of CBP are required for the repression of CLOCK/BMAL1 activity by CBP, and that this repression is not mediated by either the CRD1 or HAT domain.

### Endogenous CBP represses CLOCK/BMAL1-mediated transcription

**Figure 4:**
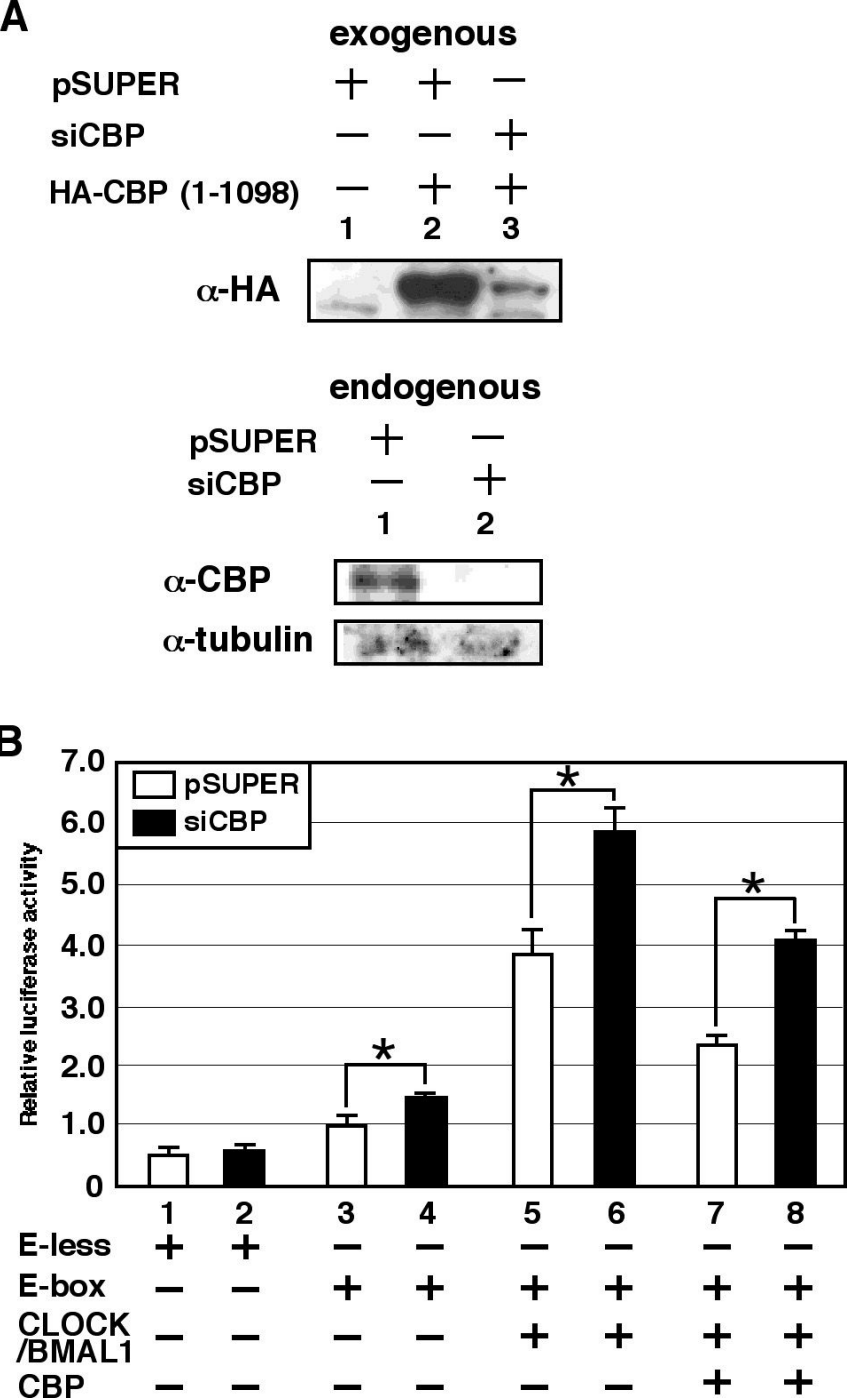
**Endogenous CBP represses CLOCK/BMAL1-mediated transcription in NIH3T3 cells**. (A) Upper panel: Cells grown in six-well plates were transiently transfected with either pSUPER or pSUPER-CBP (1.8 μg), together with either pCMV-HA or pHA-CBP1-1098 (200 ng), as indicated. Exogenous CBP was detected by Western blotting using anti-HA antibody. Lower panel: Cells grown in six-well plates were transiently transfected with either pSUPER or pSUPER-CBP (1.8 μg). Endogenous CBP was detected by Western blotting using anti-CBP antibody. α-tubulin serves as an equal loading control. (B) Cells were transiently transfected with either pE-less or pE-box (2 ng), together with either pSUPER or pSUPER-CBP (100 ng), and with or without pcDNA3CLOCK (100 ng) plus pcDNA3BMAL1 (100 ng) alone or in combination with pcDNA3CBP (50 ng), as indicated. Empty vector (pcDNA3) was used to standardize the total amount of transfected DNA (502 ng). An asterisk indicates p < 0.05 (Student's *t *test, n = 9).

The results described above are based on the overexpression of CBP. To examine the role of endogenous CBP in CLOCK/BMAL1 activity *in vivo*, we employed RNAi to reduce the expression level of endogenous CBP. RNAi expression was carried out using a pSUPER vector (pSUPER-CBP) that expressed a short hairpin RNA under the control of human H1 promoter [39], which targeted the CBP mRNA (siCBP). We first characterized the efficiency of siCBP in reducing CBP expression. As shown in Figure 4A, Western analyses revealed the nearly complete suppression of CBP expression following transfection of the cells with pSUPER-CBP but not pSUPER. It is important to note that expression of siCBP did not affect the level of forced expression of GFP (see Additional file 2). These data indicate that siCBP reduces CBP expression in NIH3T3 cells.

We next examined the effects of knockdown of CBP by RNAi on CLOCK/BMAL1-mediated transcription. Figure 4B (lane 1-4) shows that expression of siCBP significantly increased transcriptional activity of an E-box-dependent promoter, but did not affect the transcriptional activity of an E-box-less promoter. These results indicate that the increase in promoter activity by CBP knockdown is specifically mediated by the E-box sequences. Importantly, knockdown of CBP expression also significantly enhanced the activation of E-box-mediated transcription by overexpression of CLOCK and BMAL1 (Figure 4B, lane 5-6). These data strongly suggest that endogenous CBP inhibits CLOCK/BMAL1 activity. In addition, siCBP also suppressed the inhibition of CLOCK/BMAL1-mediated transcription by overexpression of CBP (Figure 4B, lane 7-8). The results in Figure 4 are consistent with our previous experiments demonstrating a role for CBP in CLOCK/BMAL1 activity using forced expression of CBP. Therefore, we conclude that CBP inhibits the activation of transcription by CLOCK/BMAL1 in NIH3T3 cells *in vivo*.

### p300 has an effect similar to CBP on CLOCK/BMAL1-mediated transcription in NIH3T3 cells

**Figure 5:**
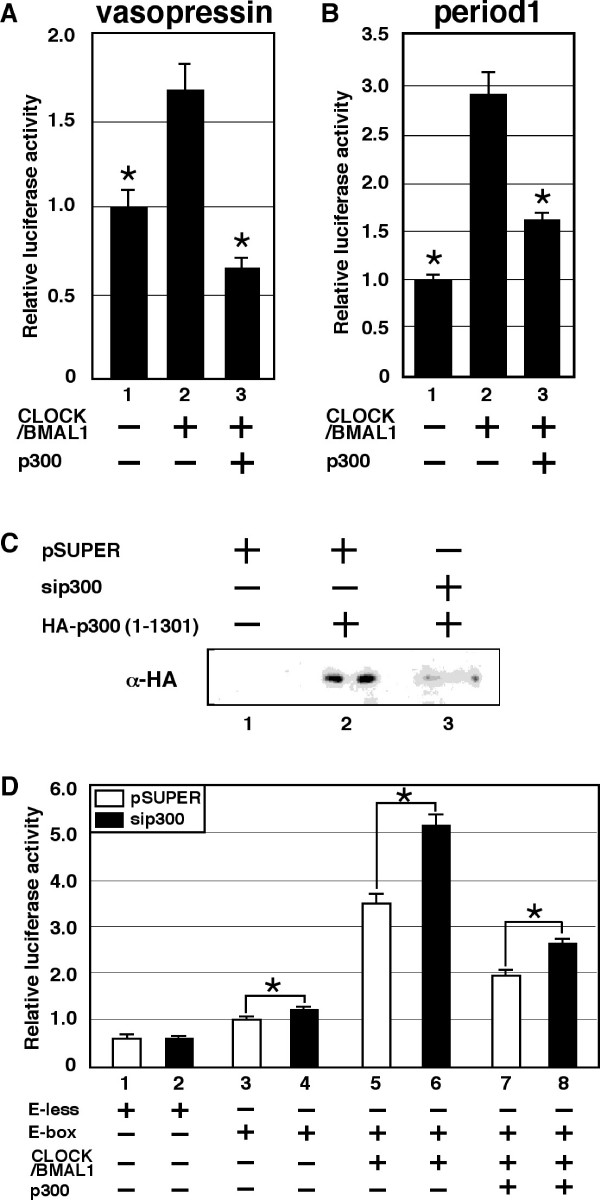
**p300 represses CLOCK/BMAL1-mediated transcription in NIH3T3 cells**. (A) pVasopressin-Luc (10 ng) or (B) pPeriod1-Luc (10 ng) were transiently transfected, with or without pcDNA3CLOCK (100 ng) plus pcDNA3BMAL1 (100 ng) alone or in combination with pcDNA3p300 (100 ng), as indicated. Empty vector (pcDNA3) was used to standardize the total amount of transfected DNA (410 ng). An asterisk indicates p < 0.05 compared with lane 2 (Student's *t *test) (C) Cells grown in six-well plates were transiently transfected with either pSUPER or pSUPER-p300 (1.8 μg), together with either pCMV-HA or pHA-p300 1-1301 (200 ng), as indicated. HA-tagged p300 was detected by Western blotting using an anti-HA antibody. (D) Cells were transfected with either pE-less or pE-box (2 ng), together with either pSUPER or pSUPER-p300 (100 ng), and with or without pcDNA3CLOCK (100 ng) plus pcDNA3BMAL1 (100 ng) alone or in combination with pcDNA3p300 (200 ng), as indicated. Empty vector (pcDNA3) was used to standardize the total amount of transfected DNA (402 ng). An asterisk indicates p < 0.05 (Student's *t *test, n = 9).

Although abundant evidence has demonstrated that p300 has an effect similar to CBP on transcriptional regulation (31-33, 40), several other reports have noted differences in the activities of p300 and CBP [41,42]. In fact, previous reports have indicated that p300, but not CBP, forms a complex with CLOCK and BMAL1 [37]. Therefore, it is possible that the effect of p300 on CLOCK/BMAL1 activity is different from that of CBP. To compare the effect of p300 on CLOCK/BMAL1-mediated transcription in NIH3T3 cells with that of CBP, we first examined the effects of overexpression of p300 in these cells. As was observed with CBP, overexpression of p300 inhibited CLOCK/BMAL1-mediated transcription from both *vasopressin *and *period1 *reporters (Figure 5A, B). To study the role of endogenous p300 in NIH3T3 cells, we examined the effects of RNAi-mediated knockdown of p300 expression on CLOCK/BMAL1-mediated transcription. As shown in Figure 5C, Western blot analyses revealed a reduction in HA-p300 1-1301 expression following transfection of cells with pSUPER-p300 but not with pSUPER. Expression of RNAi targeting p300 weakly but significantly increased transcription from an E-box-dependent promoter, without affecting transcription from an E-box-less promoter. (Figure 5D, lane1-4), leading to stimulation of CLOCK/BMAL1-mediated transcription (Figure 5D, lane 5-6). Furthermore, the inhibition of CLOCK/BMAL1-mediated transcription due to overexpression of p300 was also suppressed by knockdown of p300 expression (Figure 5D, lane 7-8). However, effects of knockdown of p300 on E-box and CLOCK/BMAL1-mediated transcription were weaker compared to effects of that of CBP (Figure 4D and 5D). These observations might reflect less inhibitory effect of p300 on CLOCK/BMAL1 activity than CBP in NIH3T3 cells.

### CBP functions as a cell type-specific positive or negative modulator of CLOCK/BMAL1 activity

**Figure 6:**
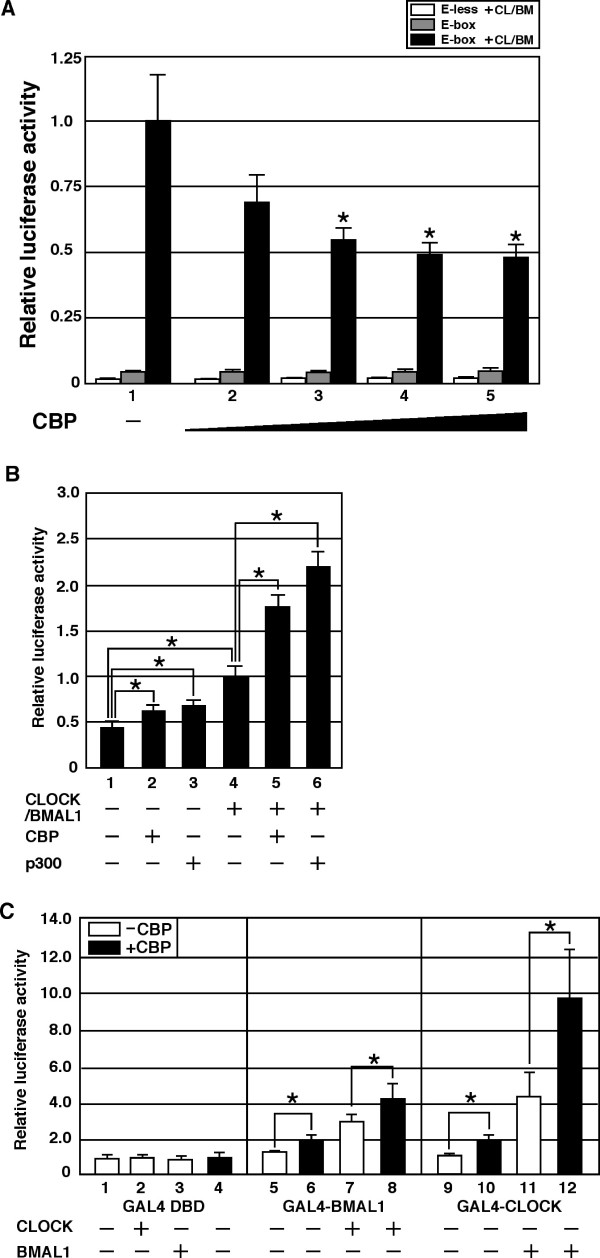
**The roles of CBP and p300 in CLOCK/BMAL1-mediated transcription in MCF7 and COS-1 cells**. (A) MCF7 cells were transiently transfected with either pE-less (white-bar) or pE-box (2 ng), together with (black-bar) or without (gray-bar) pcDNA3CLOCK (20 ng) plus pcDNA3BMAL1 (20 ng) in combination with increasing amounts of pcDNA3CBP (0, 10, 20, 50 or 100 ng), as indicated. Empty vector (pcDNA3) was used to standardize the total amount of transfected DNA (402 ng). pCMVβ (100 ng) was cotransfected as a control for transfection efficiency. An asterisk indicates p < 0.05 compared with black-bar in lane 1 (E-box + CL/BM; Student's *t *test). (B) COS-1 cells were transiently transfected with pPeriod1-Luc (10 ng), together with or without pcDNA3CLOCK (10 ng) plus pcDNA3BMAL1 (10 ng) alone or in combination with pcDNA3CBP or pCMV-p300 (100 ng), as indicated. Empty vector (pcDNA3) was used to standardize the total amount of transfected DNA (410 ng). An asterisk indicates p < 0.05 (Student's *t *test, n = 6). (C) COS-1 cells were transiently transfected with pGAL4RE-Luc (5 ng), together with either pGAL4, pGAL4-CLOCK or pGAL4-BMAL1 (100 ng) alone or in combination with pcDNA3CLOCK or pcDNA3BMAL1 (100 ng), in the presence or absence of pcDNA3CBP (100 ng), as indicated. Empty vector (pcDNA3) was used to standardize the total amount of transfected DNA (405 ng). An asterisk indicates p < 0.05 (Student's *t *test). (A, B) Luciferase activity was expressed as a ratio of CLOCK/BMAL1-mediated reporter activity.

Although the results above indicate that CBP inhibits the activity of CLOCK/BMAL1 in NIH3T3 cells, previous reports have shown that CBP enhances CLOCK/BMAL1-mediated transcription in COS-7, HEK293 and Hep3B cells [36,37]. These contrasting results raise the possibility that the effect of CBP on CLOCK/BMAL1-mediated transcription is cell type- or tissue-specific. To test this, we examined the role of CBP in CLOCK/BMAL1-mediated transcription in MCF7 and COS-1 cells under experimental conditions similar to those used in Figure 1. Previous reports showed that the circadian rhythm was observed in metabolism and proliferation in the MCF7 cells. On the other hand, COS cells have been used to investigate molecular mechanisms of regulation for CLOCK/BMAL1-mediated transcription. Coexpression of CLOCK/BMAL1 led to activation of transcription from an E-box-dependent promoter in MCF7 cells and a *period1 *promoter in COS-1 cells (Figure 6A, B). Consistent with the results in NIH3T3 cells (Figure 1A), CBP repressed CLOCK/BMAL1-mediated transcription in MCF7 cells in a dose-dependent manner (Figure 6A). However, in contrast to this result, overexpression of CBP or p300 significantly enhanced CLOCK/BMAL1-mediated transcription in COS-1 cells (Figure 6B). Additionally, overexpression of CBP or p300 stimulated the activity of the *period1 *promoter in the absence of CLOCK/BMAL1 expression (Figure 6B, lane 1-3), suggesting that p300 and CBP enhance the activity of endogenous CLOCK/BMAL1. These results indicate that CBP and p300 function as coactivators of CLOCK/BMAL1 in COS-1 cells, but as corepressors of CLOCK/BMAL1 in MCF7 cells.

The results in Figure 6A suggest differences in the mechanism of transcriptional activation by CLOCK/BMAL1 in COS-1, NIH3T3 and MCF7 cells. We therefore examined the ability of CLOCK and BMAL1 to activate transcription in COS-1 cells using fusions of these proteins with GAL4-DNA binding domain. Interestingly, in contrast to the results in NIH3T3 cells (Figure 2), GAL4-CLOCK and GAL4-BMAL1 failed to activate transcription from a GAL4-dependent promoter (Figure 6C, lane 1, 5, 9), suggesting that the function of CLOCK and BMAL1 in COS-1 cells differs from their function in NIH3T3 cells. However, overexpression of CBP enhanced the activation of transcription by GAL4-CLOCK and GAL4-BMAL1 as well as by GAL4-CLOCK/BMAL1 and GAL4-BMAL1/CLOCK heterodimers (Figure 6C, lane 5-12), supporting the observations in Figure 6B.

The results in Figure 6 suggest that both the mechanism of transcriptional activation by CLOCK/BMAL1, and the effect of CBP on CLOCK/BMAL1 function are tissue- or cell type-specific.

### Analyses of interaction between CLOCK/BMAL1 and CBP

**Figure 7:**
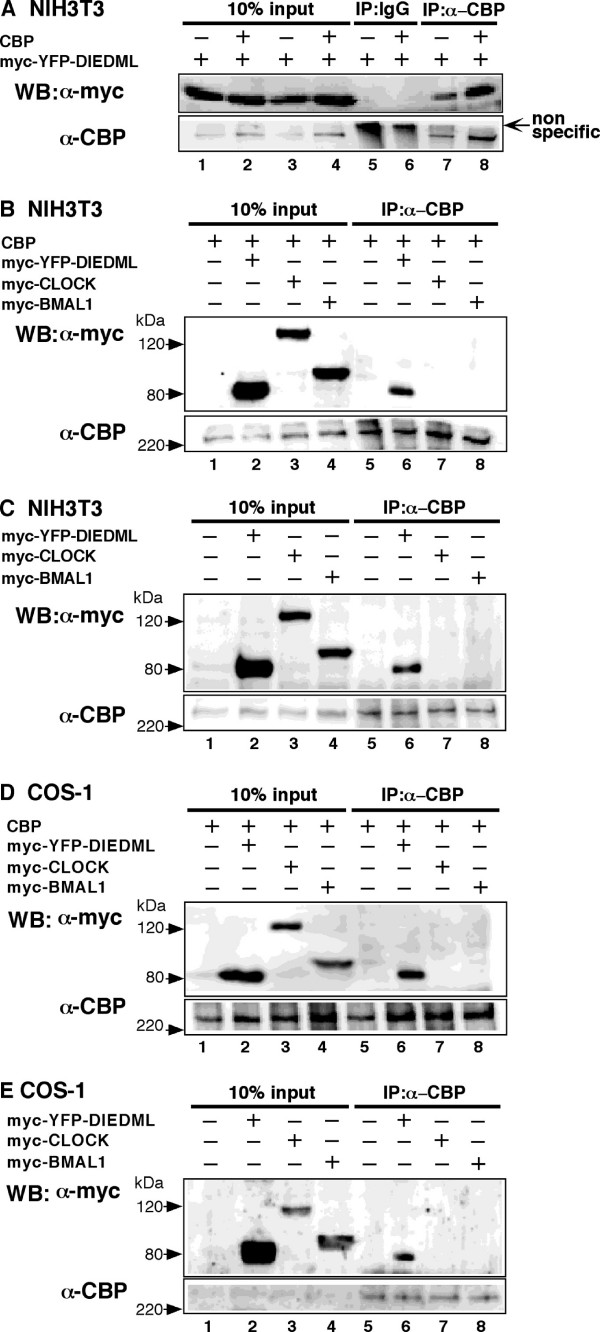
**CLOCK and BMAL1 do not coimmunoprecipitate with CBP**. (A) NIH3T3 cells were transiently transfected with pmyc-YFP-CREB_DIEDML _(1 μg), in the presence or absence of pcDNA3CBP (2 μg). Empty vector (pcDNA3) was used to standardize the total amount of transfected DNA (3 μg). (B) NIH3T3 cells were transiently transfected with pcDNA3CBP (2 μg), together with either pCMV-myc (6 μg), pmyc-CLOCK (6 μg), pmyc-BMAL1 (6 μg) or pmyc-YFP-CREB_DIEDML _(1 μg) plus pCMV-myc (5 μg). (C) NIH3T3 cells were transiently transfected with either pCMV-myc (6 μg), pmyc-CLOCK (6 μg), pmyc-BMAL1 (6 μg) or pmyc-YFP-CREB_DIEDML _(1 μg) plus pCMV-myc (5 μg). (D) COS-1 cells were transiently transfected with pcDNA3CBP (1 μg), together with either pCMV-myc (3 μg), pmyc-CLOCK (3 μg), pmyc-BMAL1 (3 μg) or pmyc-YFP-CREB_DIEDML _(0.5 μg) plus pCMV-myc (2.5 μg). (E) COS-1 cells were transiently transfected with either pCMV-myc (3 μg), pmyc-CLOCK (3 μg), pmyc-BMAL1 (3 μg) or pmyc-YFP-CREB_DIEDML _(0.5 μg) plus pCMV-myc (2.5 μg). Immunoprecipitations (IP) was performed using anti-rabbit IgG or anti-CBP antibodies, and then Western blot analysis was performed using anti-myc or anti-CBP antibodies, as indicated. The input lanes represent 10% of total cell lysates in the binding reaction.

To examine the molecular mechanism by which CBP modulates the activity of CLOCK/BMAL1, we determined whether CBP interacts with CLOCK or BMAL1 using coimmunoprecipitation and Western blot analyses. We first confirmed the interaction between CLOCK and BMAL1 under our experimental conditions. Myc-CLOCK was observed in the precipitates from lysates following incubation with the anti-HA antibody (see Additional file 1), confirming previous findings showing that CLOCK and BAML1 form a heterodimer. To test whether CBP interacts with CLOCK/BMAL1, we used myc-YFP-CREB_DIEDML _as a positive control that has been shown to constitutively interact with CBP [43]. CREB_DIEDML _is a mutant of transcription factor CREB in which six amino acids around S133 (RRPSYR) have been substituted with those from the CBP-interacting region (DIEDML) of SREBP [43]. Comparable expression levels of three myc-tagged proteins were verified by Western blot analyses using an anti-myc monoclonal antibody (Figure 7 A-E, lane 1-4; 10% input). An increase in the levels of CBP was also observed using an anti-CBP polyclonal antibody (Figure 7A, B, D). Consistent with previous reports, myc-YFP-CREB_DIEDML _was observed in the precipitates from either cell line using the anti-CBP antibody. However, myc-CLOCK and myc-BMAL1 was not observed in either of these precipitates (Figure 7B, D, but see [36,37,44]). We also examined the interactions of endogenous CBP with myc-CLOCK, myc-BMAL1 or myc-YFP-CREB_DIEDML _in NIH3T3 and COS-1 cells. Consistent with the results above, neither myc-CLOCK nor myc-BMAL1 were observed in the precipitates using the anti-CBP antibody, while myc-YFP-CREB_DIEDML _was (Figure 7C, E). These results suggest that CBP in NIH3T3 and COS-1 cells either is not bound to CLOCK or BMAL1 in our experimental conditions [but see [36,37,44]] or that CLOCK/BMAL1 exhibits a much lower affinity for CBP than does CREB_DIEDML_.

### CBP enhances the repression of CLOCK/BMAL1-mediated transcription by HDAC3

**Figure 8:**
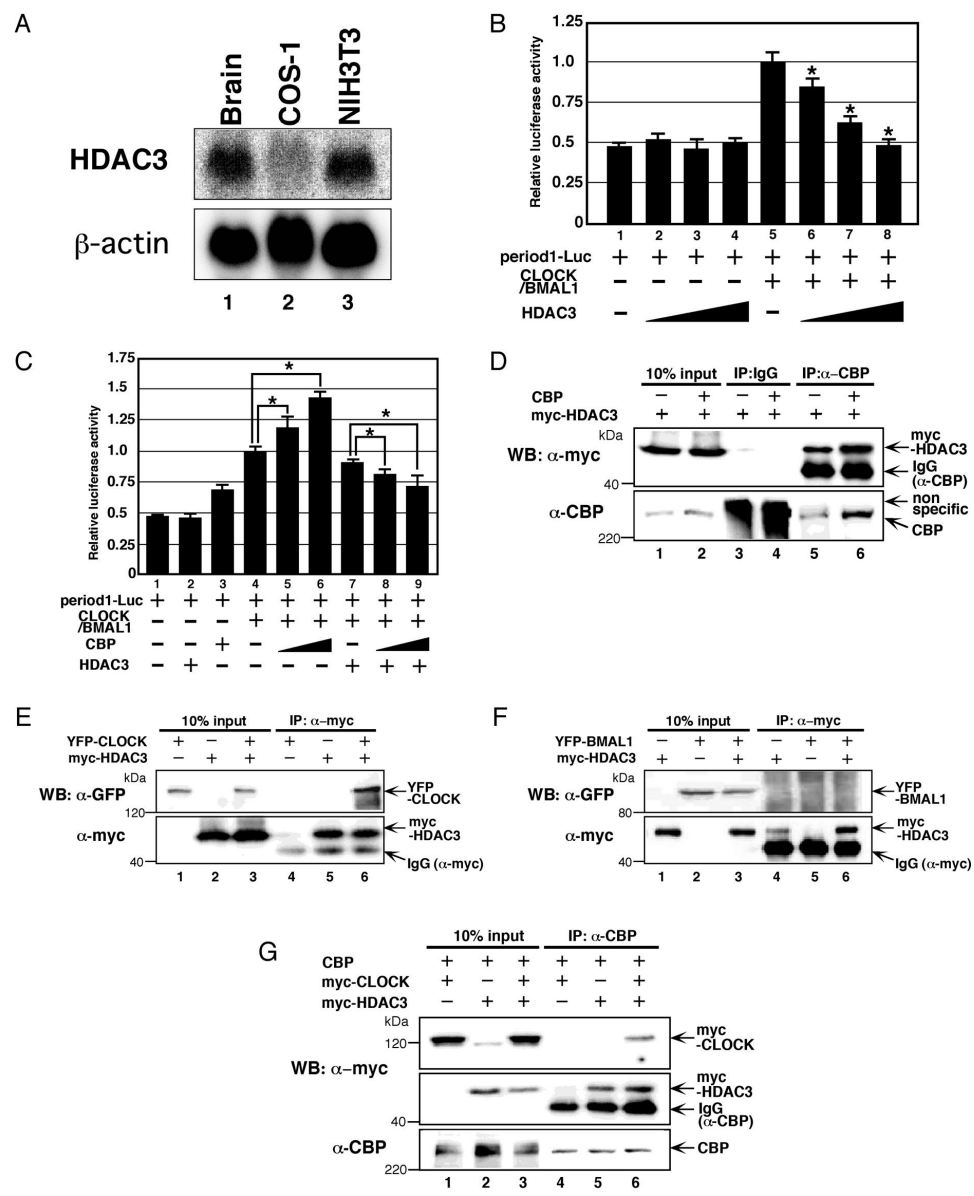
**CBP enhances the repression of CLOCK/BMAL1-mediated transcription by HDAC3**. (A) Northern blot analysis of HDAC3 mRNA expression in mouse brain, COS-1 cells and NIH3T3 cells. 30 μg (mouse brain and COS-1 cells) or 15 μg (NIH3T3 cells) of total RNA were loaded and blotted. (B) COS-1 cells were transiently transfected with pPeriod-1-Luc (10 ng), either with or without pcDNA3CLOCK (10 ng) and pcDNA3BMAL1 (10 ng), in the presence of increasing amounts (0, 20, 50, 100 ng) of pcDNA3HDAC3, as indicated. Empty vector (pcDNA3) was used to standardize the total amount of transfected DNA (210 ng). An asterisk indicates p < 0.05 compared with lane 5 (Student's *t *test). (C) pPeriod1-Luc cells were transiently cotransfected, with or without pcDNA3CLOCK (10 ng) plus pcDNA3BMAL1 (10 ng), with or without pcDNA3HDAC3 (20 ng), in combination with increasing amounts (0, 10, 30 ng) of pcDNA3CBP, as indicated. Empty vector (pcDNA3) was used to standardize the total amount of transfected DNA (210 ng). An asterisk indicates p < 0.05 (Student's *t *test). (D) COS-1 cells were transiently transfected with pmyc-HDAC3 (1 μg), in the presence or absence of pcDNA3CBP (7 μg). (E, F) COS-1 cells were transiently transfected with pEYFP-CLOCK (7 μg), pEYFP-BMAL1 (7 μg) or pmyc-HDAC3 (1 μg). Empty vector (pEYFP or pCMV-myc) was used to standardize the total amount of transfected DNA (8 μg). (G) COS-1 cells were transiently transfected with pcDNA3CBP (6 μg), in the presence or absence of pmyc-CLOCK (1 μg) or pmyc-HDAC3 (1 μg). Empty vector (pCMV-myc) was used to standardize the total amount of transfected DNA (8 μg). Immunoprecipitations (IP) was performed using anti-rabbit IgG or anti-CBP antibodies, and then Western blot analysis was performed using anti-myc, anti-CBP or anti-GFP antibodies, as indicated. The input lanes represent 10% of total cell lysates in the binding reaction. (B, C) Luciferase activity was expressed as a ratio of CLOCK/BMAL1-mediated reporter activity.

HDAC3, a transcriptional co-repressor, interacts with the N terminal region of CBP/p300 [45] that is required for repression of CLOCK/BMAL1-mediated transcription (Figure 3). In addition, we found that HDAC3 mRNA is more highly expressed in NIH3T3 than COS-1 cells (Figure 8A). Therefore, we investigated the mechanisms by which CBP functions as a co-repressor for CLOCK/BMAL1-mediated transcription by examining the effects of forced expression of HDAC3 in COS-1 cells where CBP functions as a co-activator for CLOCK/BMAL1.

We first examined the effects of forced expression of HDAC3 on CLOCK/BMAL1-mediated transcription in COS1 cells. Forced expression of HDAC3 together with CLOCK/BMAL1 inhibited CLOCK/BMAL1-mediated transcription in a dose-dependent manner (Figure 8B), indicating that HDAC3 functions as a co-repressor for CLOCK/BMAL1. We next examined the effects of forced expression of CBP on CLOCK/BMAL1-mediated transcription in the presence or absence of HDAC3 overexpression. In the absence of HDAC3 overexpression, forced expression of CBP enhanced transcriptional activation by CLOCK/BMAL1 in a dose-dependent manner, confirming previous results in Figure 6. However, in the presence of HDAC3 overexpression, co-expression of CBP enhanced the repression of CLOCK/BMAL1-mediated transcription by HDAC3 in a dose-dependent manner (Figure 8C). These results indicate that CBP can either positively or negatively modulate CLOCK/BMAL1-mediated transcription in the presence or absence of forced expression of HDACs, respectively, suggesting that the expression level of HDAC3 determines whether CBP functions as a co-activator or co-repressor.

To further examine the molecular mechanisms responsible for the inhibition of the activity of CLOCK/BMAL1 by HDAC3 and CBP, we tested whether HDAC3, CBP and CLOCK form a complex in COS-1 cells. Myc-HDAC3 was co-precipitated by an anti-CBP antibody but not the control IgG-antibody (Figure 8D). Importantly, we observed that forced expression of CBP resulted in increased precipitation of myc-HDAC3. These results confirm the previous finding that CBP interacts with HDAC3. YFP-CLOCK (Figure 8E) but not YFP-BMAL1 (Figure 8F) was found in anti-myc precipitates when myc-HDAC3 was co-expressed, suggesting that CLOCK interacts with HDAC3. Finally, myc-CLOCK and myc-HDAC3 were co-precipitated by anti-CBP antibody when myc-CLOCK and myc-HDAC3 were co-expressed (Figure 8G). However, myc-CLOCK was co-precipitated by the anti-CBP antibody only when myc-HDAC3 was co-expressed. Thus, these results are consistent with our observation that CBP does not interact directly with CLOCK and suggest that CBP forms a complex with CLOCK through interaction with HDAC3, functioning as a corepressor for CLOCK/BMAL1.

### CBP enhances activation of CLOCK/BMAL1-mediated transcription by pCAF

**Figure 9:**
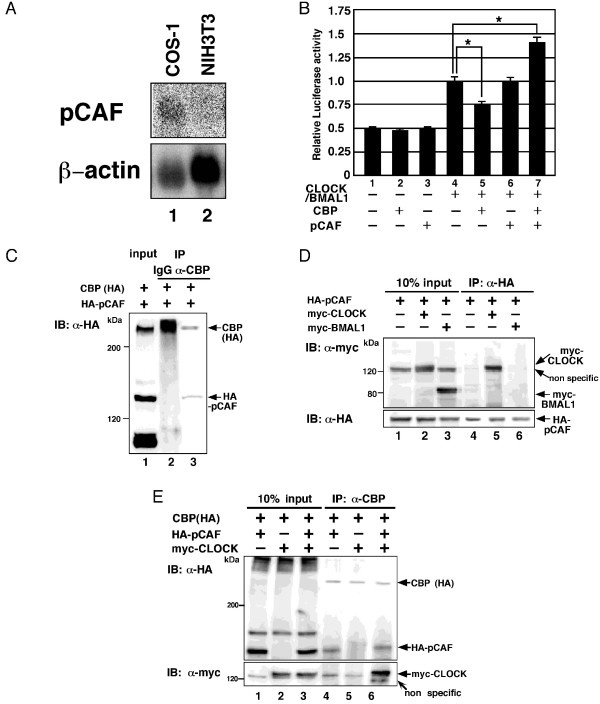
**CBP enhances activation of CLOCK/BMAL1-mediated transcription by pCAF**. (A) Northern blot analysis of HDAC3 mRNA expression in COS-1 cells and NIH3T3 cells. 30 μg of total RNA were loaded and blotted. (B) NIH3T3 cells were transiently transfected with pPeriod-1-Luc (10 ng), either with or without pcDNA3CLOCK (100 ng), pcDNA3BMAL1 (100 ng), pcDNA3CBP (50 ng), and pcDNA3pCAF (50 ng), as indicated. Empty vector (pcDNA3) was used to standardize the total amount of transfected DNA (410 ng). Luciferase activity was expressed as a ratio of CLOCK/BMAL1-mediated reporter activity. An asterisk indicates p < 0.05 (Student's *t *test). (C) COS-1 cells were transiently transfected with pHA-pCAF (2 μg), in the presence of pcDNA3CBP (6 μg). (D) COS-1 cells were transiently transfected with pmyc-CLOCK (1 μg), pmyc-BMAL1 (1 μg), or pHA-pCAF (7 μg). Empty vector (pCMV-myc or pCMV-HA) was used to standardize the total amount of transfected DNA (8 μg). (E) COS-1 cells were transiently transfected with pcDNA3CBP (6 μg), in the presence or absence of pmyc-CLOCK (0.5 μg) or pHA-pCAF (1.5 μg). Empty vector (pCMV-myc or pCMV-HA) was used to standardize the total amount of transfected DNA (8 μg). Immunoprecipitations (IP) were performed using anti-rabbit IgG, anti-CBP, or anti-HA antibodies, and then Western blot analysis was performed using anti-CBP, anti-myc, or anti-HA antibodies, as indicated. The input lanes represent 10% of total cell lysates in the binding reaction.

It is possible that CBP requires tissue-specific cofactors to stimulate CLOCK/BMAL1-mediated transcription, similar to the findings in Figure 8. A previous report indicated that CLOCK directly interacts with the transcriptional co-activator pCAF [44]. Additionally, we found that pCAF mRNA is more highly expressed in COS cells than in NIH3T3 cells (Figure 9A). Therefore, we examined the effects of forced expression of pCAF on modulation of CLOCK/BMAL1-mediated transcription by CBP in NIH3T3 cells where CBP normally represses CLOCK/BMAL1-mediated transcription. Consistent with previous observations, forced co-expression of CBP repressed the transcriptional activation of CLOCK/BMAL1 in the absence of pCAF expression. However, when pCAF was overexpressed, forced expression of CBP enhanced transcriptional activation by CLOCK/BMAL1 although forced expression of pCAF alone did not affect CLOCK/BAML1-mediated transcription (Figure 9B). These data suggest that CBP functions as a co-activator for CLOCK/BMAL1 only when pCAF is highly expressed.

To further address the mechanisms by which CBP enhances CLOCK/BMAL1-mediated transcription in the presence of pCAF overexpression, we performed co-immunoprecipation assays. Consistent with previous observations, HA-pCAF was coprecipitated by the anti-CBP antibody but not the IgG-antibody (Figure 9C) [46], indicating that CBP interacts with pCAF. Myc-CLOCK but not myc-BMAL1 was coprecipitated by anti-HA-antibody when HA-pCAF was co-expressed (Figure 9D), indicating that pCAF interacts with CLOCK but not BMAL1. Finally, both HA-pCAF and myc-CLOCK were coprecipitated by anti-CBP antibody when myc-CLOCK and HA-pCAF were co-expressed. However, mycCLOCK was not precipitated by anti-CBP antibody when pCAF was not co-expressed (Figure 9E). Similar to the case of HDAC3, these data indicate that CBP forms a complex with CLOCK through interacting with pCAF, and functions as a co-activator for CLOCK/BMAL1.

## Discussion

Previous reports have demonstrated that overexpression of CBP or p300 stimulates the activation of transcription by the CLOCK/BMAL1 heterodimer in COS-7, Hep3B and HEK293 cells [36,37]. Consistent with this, we observed that CBP enhances CLOCK/BMAL1-mediated transcription in COS-1 cells (Figure 6B). These findings suggest that CBP and p300 function as coactivators for CLOCK/BMAL1. However, in NIH3T3 cells, both CBP and p300 inhibited CLOCK/BMAL1-mediated transcription from both an E-box-containing reporter and the *vasopressin *and *period1 *promoters (Figure 1A, C, D). In addition, knockdown of CBP or p300 expression by RNAi leads to significant enhancement of CLOCK/BMAL1-mediated transcription (Figure 4B, 5D), indicating that CBP and p300 repress CLOCK/BMAL1 activity in NIH3T3 cells. Similarly, CBP also repressed CLOCK/BMAL1 activity in MCF7 cells (Figure 6A). These opposing effects of CBP and p300 on CLOCK/BMAL1-mediated transcription in different types of cultured cells suggest that CBP/p300 acts as a tissue-specific positive or negative modulator of CLOCK/BMAL1 activity.

A previous report using NIH3T3 cells showed that CBP functions as a co-activator for CLOCK/BMAL1-mediated transcription from a synthetic E-box-dependent promoter (pM34-Luc) different from that (pE-box) used in this study [44]. Indeed, the sequences neighbouring the pM34-Luc and pE-boxes are different [13,14]. Under our experimental conditions, we confirmed previous observations that forced coexpression of CBP enhanced the activation of transcription from pM34-Luc by CLOCK/BMAL1 in NIH3T3 cells (see Additional file 3). This finding suggests that the effects of CBP on CLOCK/BMAL1-mediated transcription are dependent on the promoter context, including the sequence surrounding an E-box [47,48].

In the present study, we hypothesized that the positive or negative modulating function of CBP on CLOCK/BMAL1 reflects the level of expression of tissue-specific cofactors such as pCAF and HDAC3 that are highly expressed in COS-1 and NIH3T3 cells, respectively. We found that CBP enhanced CLOCK/BMAL1-mediated transcription when pCAF was overexpressed in NIH3T3 cells, whereas it normally represses the activity of CLOCK/BMAL1. Conversely, CBP repressed CLOCK/BMAL1-mediated transcription when HDAC3 was overexpressed in COS-1 cells, in which it normally enhances the activity of CLOCK/BMAL1. Interestingly, results of co-immunoprecipitation assays indicated that CBP forms a complex with CLOCK and HDAC3 or pCAF and that CBP and CLOCK complex formation is indirect and mediated by the interaction with HDAC3 or pCAF (but see [36,37,44]). Consistent with this, CBP repressed or enhanced the activation of transcription by GAL4-CLOCK in NIH3T3 and COS-1 cells, respectively, suggesting that CBP targets CLOCK in both cell lines. Thus, our findings strongly suggest that CBP functions as a co-activator or co-repressor for CLOCK/BMAL cooperatively with pCAF or HDAC3, respectively.

**Figure 10:**
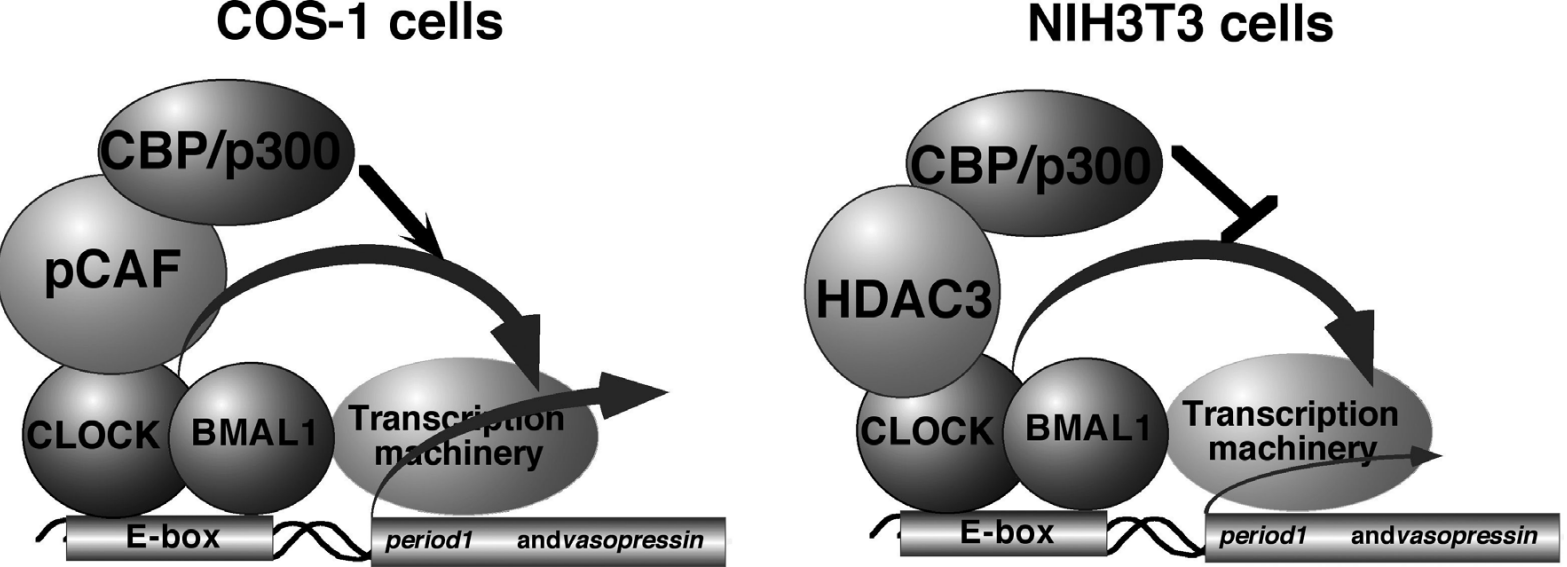
**Possible mechanisms for positive or negative modification of CLOCK/BMAL1-mediated transcription by CBP**. (Left) CBP functions as a coactivator of CLOCK/BMAL1 through an interaction with co-activator pCAF. (Right) CBP functions as a corepressor of CLOCK/BMAL1 through an interaction with co-repressor HDAC3.

CBP functions as a co-repressor for CLOCK/BMAL1 through the formation of complexes with CLOCK and HDAC3. In agreement with our findings, several reports have shown that CBP and p300 act as co-repressors by cooperating with other transcriptional regulators [33-35]. Indeed, the CRD1 domain of CBP has been shown to mediate strong repression of transcription through interactions with HDAC6 [35]. CRD1 is covalently modified by small ubiquitin-like modifier-1 (SUMO-1) [35]. SUMO-modified CRD1 binds HDAC6 and leads to transcriptional repression [35]. Additionally, CBP functions as a co-repressor by acetylating the transcriptional activator, *Drosophila *T-cell factor (dTCF) [34], as well as histones [32,33]. The acetylation of dTCF by a HAT domain of CBP lowers the affinity of dCTF for Armadillo, which functions as a co-activator for dCTF, and leads to the repression of transcription [34]. These previous findings suggest that covalent modification of CBP or by CBP plays key roles in the mechanisms by which CBP functions as a co-repressor. It will be important to investigate the mechanisms of complex formation between CLOCK/BMAL1, CBP and HDAC3 or pCAF in detail (Figure 10).

Regulation of the circadian transcriptional rhythm of core clock genes by CLOCK/BMAL1 has been observed in both the SCN and peripheral tissues [1,2,11,15,16,18,21]. Importantly, although transcriptional rhythms regulated by CLOCK/BMAL1 are similar in different tissues, minor differences are apparent in the waveform and amplitude of these transcriptional rhythms [6,22,23]. In addition, the level of expression of CLOCK/BMAL1 target genes differs between tissues [6,18,14-26]. Our observation that the general co-activator CBP/p300 and tissue-specific cofactors HDAC3 and pCAF cooperatively modify CLOCK/BMAL1 activity suggests that they might contribute to the differences in the transcriptional rhythm and expression level of CLOCK/BMAL1 target genes in different tissues, modulating cell type-specific functions such as metabolisms, homeostasis and neural plasticity at the cellular level. A recent study showed that CBP participates in transcriptional regulation by *Drosophila *CLOCK/CYCLE heterodimer [49], supporting our findings. Genetic studies are required to investigate the *in vivo *roles of circadian rhythmicity of these transcriptional cofactors.

## Conclusion

Our findings indicate possible mechanisms by which CBP/p300 tissue-specifically acts cooperatively with pCAF and HDAC3 either as a co-activator or co-repressor, respectively, for CLOCK/BMAL1.

## Methods

### Plasmid construction

A detailed description of all plasmids can be found in the see Additional file 4.

### Cell culture and transient transfection

NIH3T3 and COS-1 cells were maintained as previously described [50]. MCF7 cells were maintained in DMEM supplemented with 10% FBS, penicillin (100 U/ml), streptomycin (100 mg/ml) and insulin (60 ng/ml) at 37°C in 5% CO_2_. Cells were transiently transfected using the Lipofectamin-plus reagent (Invitrogen, Carlsbad, CA) according to the manufacturer's instructions.

### Reporter assay

NIH3T3 and MCF7 cells were grown in 24-well plates and COS-1 cells in 12-well plates. Forty-eight hrs following transfection, we measured activities of luciferase and β-galactosidase as previously described [50]. For assays except for Fig. 6A, pCH110 (100 ng) was used as a control for transfection efficiency. Luciferase activity was normalized to β-galactosidase activity and expressed as a ratio of CLOCK/BMAL1-mediated reporter activity. All reporter assays were performed in triplicate in three independent experiments. Each value represents the mean +/- SD.

### Immunoprecipitation and Western blotting

NIH3T3 cells grown in 100-mm dishes and COS-1 cells grown in 60-mm dishes were transiently transfected with different expression vectors as indicated in Figure legends. Immunoprecipitation and Western blotting were performed as previously described [50]. Polyclonal anti-CBP antibody (A-22; SantaCruz, Biotechnology, Santa Cruz, CA), anti-myc antibody (Clontech, Mountain View, NJ), anti-HA antibody (Roche Molecular Biochemicals, Mannheim, Germany) and anti-rabbit IgG were used for immunoprecipitation. Western blotting membranes were probed with anti-CBP antibody (1:1000), anti-HA antibody (1:1000), anti-myc antibody (1:1000), anti-GFP antibody (598; 1:1000, MBL, Japan), and anti-α-tubulin (sc-5286; 1:1000, SantaCruz) as primary antibodies and then visualized with peroxidase-conjugated anti-mouse or anti-rabbit IgG (1:1000, SantaCruz) as secondary antibodies.

### RNAi

To silence the expression of CBP, p300, and GFP, we targeted sequences in the middle of the open reading frames of CBP (AACAGTGGGAACCTTGTTCCA, nucleotides 160 to 180), p300 (AATTGGGACTAACCAATGGTG, nucleotides 155 to 175) and EGFP (AAGCTGACCCTGAAGTTCATC, nucleotides 124 to 144). As described above, pSUPER vectors were transiently transfected into cells which were analyzed 60 hrs later.

### Northern blotting

Isolation of total RNAs and Northern blot analysis were performed as previously described [51]. Full-length cDNAs for HDAC3 and pCAF were used as probes.

## Abbreviations

BMAL1: brain-muscle-arnt-like-protein 1; CREB: cAMP response element binding protein; CBP: CREB binding protein; pCAF: p300/CBP associate factor; HDAC: histone deacetylase; HAT: histone acetyltransferase; SCN: suprachiasmatic nuclei; GFP: green fluorescent protein; mRNA: messenger ribonucleic acid; RNAi: RNA interference; siRNA: small interfering RNA.

## Competing interests

The authors declare that they have no competing interests.

## Authors' contributions

HH participated in the overall design of the study and in drafting the manuscript, and performed all experiments and the statistical analysis. HA, MI and HK participated in the cloning of the expression plasmids and design and performance of the two-hybrid assays. KK, TI and AS participated in the cloning of the expression plasmids and design and performance of the immuno-precipitation and western blotting. SM and SK participated in the overall design of the study. SK wrote the manuscript. All authors read and approved the final manuscript.

## Supplementary Material

Additional file 1 **Interaction of CLOCK and BMAL1 in NIH3T3 cells**. NIH3T3 cells were transiently transfected with pHA-BMAL1 (7 μg) or pmyc-CLOCK (1 μg). Empty vector (pCMV-HA or pCMV-myc) was used to standardize for the total amount of transfected DNA (8 μg). Immunoprecipitations (IP) were performed using anti-HA antibodies, and then Western blot analysis was performed using anti-HA or anti-myc antibodies, as indicated. The input lanes represent 10% of total cell lysate in the binding reaction. Click here for file

Additional file 2 **Transiently transfected pSUPER-CBP did not affect the protein levels of GFP**. Cells grown in six-well plates were transiently transfected with either pSUPER, pSUPER-CBP (1.8 μg) or pSUPER-GFP, together with either pCMV-HA, pHA-CBP 1-1098 (200 ng) or pmyc-YFP-CREB_DIEDML _(200 ng), as indicated. Exogenous CBP or myc-YFP-CREB_DIEDML _was detected by Western blotting using anti-HA or anti-myc antibody, as indicated. Click here for file

Additional file 3 **Forced coexpression of CBP enhanced CLOCK/BMAL1-mediated transcription of pM34-Luc**. Cells were transiently transfected with either pM34-Luc or pE-box (2 ng), either with or without pcDNA3CLOCK (30 ng) and pcDNA3BMAL1 (30 ng), in combination with pcDNA3CBP (50 ng), as indicated. Empty vector (pcDNA3) was used to standardize for total amount of transfected DNA (502 ng). Luciferase activity was expressed as a ratio of CLOCK/BMAL1-mediated reporter activity. An asterisk indicates p < 0.05 (Student's *t *test). Click here for file

Additional file 4 **Plasmid constructions**. Word document detailing the plasmid constructions. Click here for file
