# Supplementary material for: CBP/p300 is a cell type-specific modulator of CLOCK/BMAL1-mediated transcription
Source: Mol Brain. 2009 Nov 19;2:34. doi: 10.1186/1756-6606-2-34 (PMC2785803; doi:10.1186/1756-6606-2-34)
Supplement: Additional file 1 — Interaction of CLOCK and BMAL1 in NIH3T3 cells. NIH3T3 cells were transiently transfected with pHA-BMAL1 (7 μg) or pmyc-CLOCK (1 μg). Empty vector (pCMV-HA or pCMV-myc) was used to standardize for the total amount of transfected DNA (8 μg). Immunoprecipitations (IP) were performed using anti-HA antibodies, and then Western blot analysis was performed using anti-HA or anti-myc antibodies, as indicated. The input lanes represent 10% of total cell lysate in the binding reaction. [file 1756-6606-2-34-S1.PDF]

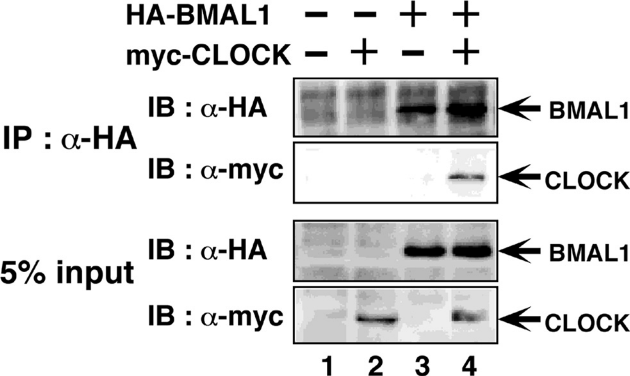
HA-BMAL1 − − + +
myc-CLOCK − + − +
IP : α-HA
IB : α-HA
BMAL1
IB : α-myc
CLOCK
5% input
IB : α-HA
BMAL1
IB : α-myc
CLOCK
1 2 3 4
