# Supplementary figures and images for: CBP/p300 is a cell type-specific modulator of CLOCK/BMAL1-mediated transcription
Source: Mol Brain. 2009 Nov 19;2:34. doi: 10.1186/1756-6606-2-34 (PMC2785803; doi:10.1186/1756-6606-2-34)

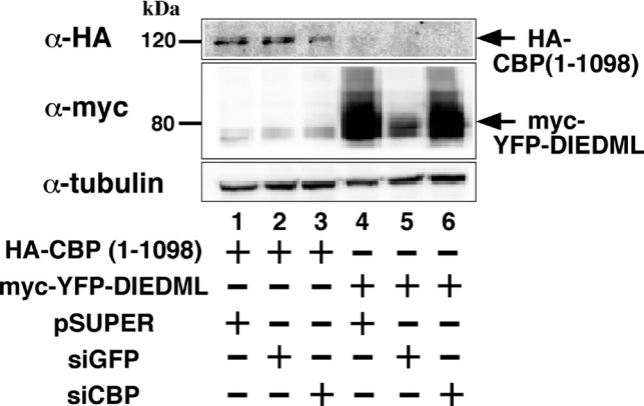

Supplement: Additional file 2 — Transiently transfected pSUPER-CBP did not affect the protein levels of GFP. Cells grown in six-well plates were transiently transfected with either pSUPER, pSUPER-CBP (1.8 μg) or pSUPER-GFP, together with either pCMV-HA, pHA-CBP 1-1098 (200 ng) or pmyc-YFP-CREBDIEDML (200 ng), as indicated. Exogenous CBP or myc-YFP-CREBDIEDML was detected by Western blotting using anti-HA or anti-myc antibody, as indicated. [file 1756-6606-2-34-S2.PDF]

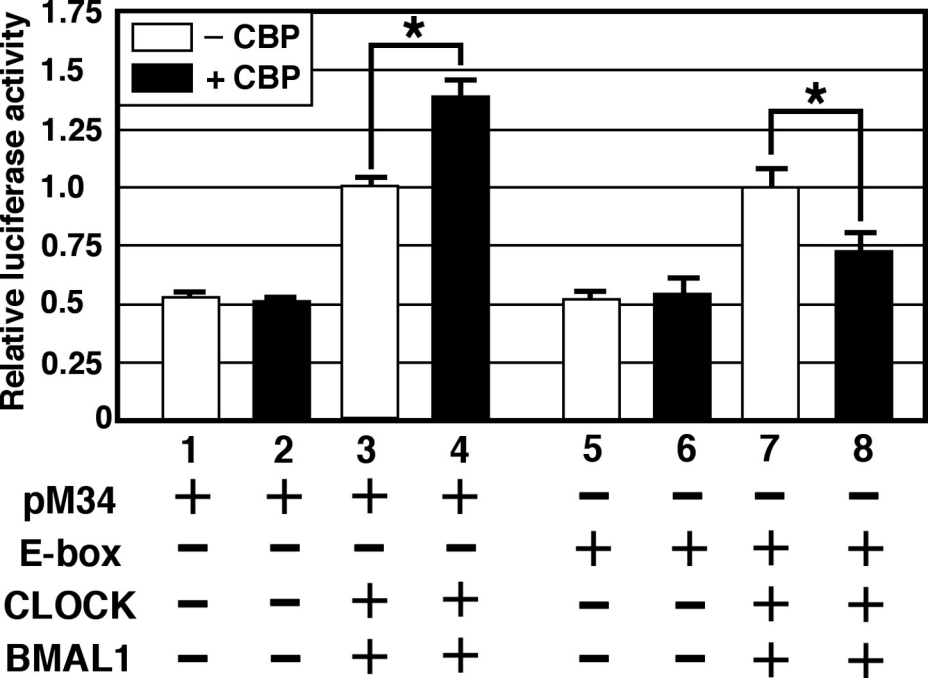

Supplement: Additional file 3 — Forced coexpression of CBP enhanced CLOCK/BMAL1-mediated transcription of pM34-Luc. Cells were transiently transfected with either pM34-Luc or pE-box (2 ng), either with or without pcDNA3CLOCK (30 ng) and pcDNA3BMAL1 (30 ng), in combination with pcDNA3CBP (50 ng), as indicated. Empty vector (pcDNA3) was used to standardize for total amount of transfected DNA (502 ng). Luciferase activity was expressed as a ratio of CLOCK/BMAL1-mediated reporter activity. An asterisk indicates p < 0.05 (Student's t test). [file 1756-6606-2-34-S3.PDF]
