# Supplementary material for: CBP/p300 is a cell type-specific modulator of CLOCK/BMAL1-mediated transcription
Source: Mol Brain. 2009 Nov 19;2:34. doi: 10.1186/1756-6606-2-34 (PMC2785803; doi:10.1186/1756-6606-2-34)
Supplement: Additional file 4 — Plasmid constructions. Word document detailing the plasmid constructions. [file 1756-6606-2-34-S4.DOC]

Additional file 4- Additional method
File format: DOC

Title: Plasmid constructions

**Plasmid constructions**

E-box-dependent luciferase reporter plasmids (pE-box), containing an E-box-dependent synthetic promoter harboring three copies of the E-box sequence from the mouse *vasopressin* promoter (positions -143 to -160), were constructed as previously described (1). E-box-dependent luciferase reporter plasmids (pM34-Luc), containing an E-box-dependent synthetic promoter harboring three copies of the E-box sequence (the CLOCK:BMAL1 M34 core binding site 5’-TGGACACGTGACCC-3’), were constructed as previously described (2). A 200 bp fragment containing the transcription initiation site from the mouse *vasopressin* promoter (positions -170 to +30), and a 3464 bp fragment containing the transcription initiation site from the mouse *period1* promoter (positions –1772 to +1692) were obtained by PCR using mouse genomic DNA as a template, and the resulting PCR fragments were subcloned into the *Kpn*I-*Nco*I sites in pGL3 (Promega, Madison, WI), generating pVasopressin-Luc and pPeriod1-Luc, respectively. A 305 bp fragment containing five copies of the GAL4 response element (GAL4RE) and the SV40 minimal promoter was amplified by PCR using pG5CAT vector (Clontech, Mountain View, NJ) as a template, and the resulting PCR fragment was subcloned into the *Mlu*I-*Nco*I sites of pGL3, generating pGAL4-Luc. The CRE-dependent luciferase reporter plasmid, pCRE-Luc, was purchased from Stratagene (La Jolla, CA). The β-galactosidase expression plasmids, pCH110 and pCMVβ, were purchased from Amersham (Piscataway, NJ) or Clontech, respectively.

pBS–CLOCK, pBS-BMAL1, pcDNA3 CLOCK, pcDNA3 BMAL1 and pmyc CLOCK, were constructed as previously described (3). Full-length of CLOCK and BMAL1 were obtained by reverse transcriptase (RT)-PCR using brain mRNA of C57BL/6N mice as a template. To generate pGAL4-CLOCK, the *Bam*HI-*Xho*I fragment encoding CLOCK from pBS-CLOCK was subcloned into the *Bam*HI-*Sal*I sites in pM (pGAL4; Clontech). To generate pEYFP-CLOCK, the *BspE*I-*Xho*I fragment encoding CLOCK from pBS-CLOCK was subcloned into the *BspE*I-*Sal*I sites pEYFP-C1 (Clontech). To generate pmyc-BMAL1 and pHA-BMAL1, the *Sal*I-*Not*I fragment encoding BMAL1 from pBS-BMAL1 was subcloned into the corresponding restriction sites in pCMV-myc (pmyc; Clontech) and pCMV-HA (pHA; Clontech), respectively. To generate pGAL4-BMAL1, the *Eco*RI-*Xba*I fragment encoding BMAL1 from pBS-BMAL1 was subcloned into the corresponding restriction sites in pM. A full-length cDNA encoding rat CREB was amplified by PCR using RSV-CREB as a template, as previously described (4), and the resulting PCR fragment encoding CREB was subcloned into the *Hin*dIII-*Bam*HI sites of pcDNA3, generating pcDNA3CREB. To generate the CREB_DIEDML_ mutant (5), two separate fragments of CREB cDNA (nucleotides 1 to 408 and 406 to 1026) were amplified by PCR using the following primers: 1/408 forward primer, GGGAAGCTTGCCGCCACCATGACGCGTAGATCTATGACCATGGACTCTGGA and 1/408 reverse primer, GGGGGATCCTCC**CTTAAG**CATGTCTTCTATGTCTGAAAGGATTTGCCTTCG (Mutated sequences are underlined); 406/1026 forward primer, GGGAAGCTT**CTTAAG**ATTTTGAATGATTTATCA and 406/1026 reverse primer, GGGGGATCCACGCGTGAATTCTTAATCTGACTTGTGGCAGTAAAGGTCTTTAAGTGCTTTTAGCTCC. To ligate the two fragments, we made use of *Afl*II restriction sites that we had introduced at the 3’ and 5’ ends of the coding region by amplification with the above primers (see sequences in bold). We also had introduced a mutation from C to T to eliminate the endogenous *Afl*II site (see sequence in box). Modification of the sequence to encode these restriction sites did not alter the final amino acid sequence of the ligated fragments encoding the CREB_DIEDML_ mutant. The cDNA encoding the full length CREB_DIEDML_ mutant comprising the two ligated PCR fragments was inserted into the *Hin*dIII-*Bam*HI sites of pBluescript, generating pBS-CREB_DIEDML_. A cDNA encoding YFP was amplified by PCR using pEYFP-C1 as the template, and the resulting PCR fragment was subcloned into the *Sfi*I-*Not*I sites in pCMV-myc, generating pmyc-YFP. To generate pmyc-YFP-CREB_DIEDML_, the *Bgl*II-*Eco*RI fragment encoding the CREB_DIEDML_ mutant from pBS-CREB_DIEDML_ was subcloned into the corresponding restriction sites in pmyc-YFP.

RSV-human CBP was kindly provided by Dr. S.H. Goodman (6). To generate pcDNA3 CBP, the *Hin*dIII -*Xba*I fragment of CBP from RSV-CBP was subcloned into the corresponding restriction sites in pcDNA3 (Invitrogen, Carlsbad, CA). To generate pGAL4-CBP, a *Sma*I-*Xba*I fragment of CBP from pcDNA3CBP was subcloned into *Eco*RI (blunt-end) -*Xba*I sites in pM, which had been digested with *Eco*RI and blunted with T4 DNA polymerase prior to digest with *Xba*I. A series of deletion mutants of the cDNA encoding CBP were cloned into appropriate restriction sites in pCMV-HA. These included a 3307 bp blunt-ended *Sma*I-*Xba*I fragment from pcDNA3CBP encoding amino acids 1-1098 of CBP, a 967 bp *Eco*RV fragment encoding aa 1-312, a 2142 bp blunt-ended *Bam*HI-*Sph*I fragment encoding aa 1-711, a 3311 bp blunt-ended *Sma*I -*Xba*I fragment encoding aa 1-1098, a 2737 bp blunt-ended *Stu*I-*Xba*I fragment encoding aa 185-1098, a 2358 bp blunt-ended *Eco*RV-*Xba*I fragment encoding aa 313-1098, a 3975 bp *Xba*I (blunt-end)-*Bam*HI fragment encoding aa 1099-2441, a 2308 bp blunt-ended *Xba*I-*Nae*I fragment encoding aa 1099-1867 and a 1669 bp *Nae*I-*Bam*HI fragment encoding aa 1868-2441. A 1157 bp blunt-ended *Sph*I-*Xba*I fragment encoding CBPΔKIX (Δ452-714) from pcDNA3CBP was ligated to a 6805 bp blunt-ended *Eco*RI-*Xba*I fragment from pcDNA3CBP1-1098, generating pcDNA3 CBPΔKIX. To generate pHA -CBPΔKIX, a 340 bp *Eco*RI-*Eco*RV fragment encoding CBPΔKIX from pcDNA3 CBPΔKIX was subcloned into the corresponding restriction sites in pHA-CBP1-1098. A full-length cDNA encoding p300 was synthesized by PCR using a human cDNA library (Clontech) as a template, and the PCR product was then subcloned into the *Hin*dIII-*Not*I sites in pcDNA3, generating pcDNA3 p300. To generate pHA-p300 and pHA-p300 1-1301, the *Sal*I-*Not*I fragment encoding full-length p300 or blunt-ended *Hin*dIII-*Xba*I fragment for p300 (aa 1-1301), respectively, from pcDNA3p300 was subcloned into the corresponding restriction sites in pCMV-HA.

Full-length cDNAs encoding murine HDAC3 and pCAF were obtained by reverse transcriptase (RT)-PCR using brain mRNA of C57BL/6N mice as a template, and the resulting PCR fragments was subcloned into the *Bam*HI-*Xba*I sites of pBluescript II (SK-) (Stratagene), generating pBS-HDAC3 and pBS-pCAF. A *Bam*HI-*Xba*I fragment encoding HDAC3 from pBS-HDAC3 or pCAF from pBS-pCAF were subcloned into the corresponding restriction sites in pcDNA3, generating pcDNA3 HDAC3 and pcDNA3 pCAF, respectively. To generate pmyc-HDAC3 and pHA-pCAF, a *BamH*I-*Not*I fragment encoding HDAC3 from pBS-HDAC3 or pCAF from pBS-pCAF were subcloned into the *Bgl*II-*Not*I site in pmyc and pHA, respectively.

To generate pSUPER-CBP, pSUPER-300, and pSUPER-GFP, we performed PCR using pSUPER (7) as a template using the following primers: pSUPER-CBP forward primer, GAGATGGAACAAGGTTCCCACTGTTTTTGGAAAAGCTTATCGATACCG, pSUPER-CBP reverse primer, TTGAATGGAACAAGGTTCCCACTGGGGATCTGTGGTCTCATACAG, pSUPER-p300 forward primer, GAGACACCATTGGTTAGTCCCAATTTTTGGAAAAGCTTATCGATACCG, pSUPER-p300 reverse primer, TTGAACACCATTGGTTAGTCCCAAGGGATCTGTGGTCTCATACAG, pSUPER-GFP forward primer, GAGAGATGAACTTCAGGGTCAGCTTTTTGGAAAAGCTTATCGATACCG, pSUPER-GFP reverse primer, TTGAAGATGAACTTCAGGGTCAGCGGGATCTGTGGTCTCATACAG.　The resultant PCR fragment was self-ligated to generate a pSUPER vector containing the target sequence of CBP, p300 or GFP.

**References**:

1. Hogenesch JB, Gu YZ, Jain S, Bradfield CA: **The basic-helix-loop-helix-PAS orphan MOP3 forms transcriptionally active complexes with circadian and hypoxia factors.** *Proc Natl Acad Sci USA* 1998, **95:** 5474-5479.

2. Jin X, Shearman LP, Weaver DR., Zylka MJ, De Vries GJ, Reppert SM: **A molecular mechanism regulating rhythmic output from the suprachiasmatic circadian clock.** *Cell* 1999, 96: 57-68.

3. Hosoda H, Motohashi J, Kato H, Masushige S, Kida S: **A BMAL1 mutant with arginine 91 substituted with alanine acts as a dominant negative inhibitor.** *Gene* 2004, **338:** 235-241.

4. Kida S, Josselyn SA., de Ortiz SP, Kogan JH., Chevere I, Masushige S, Silva AJ: **CREB required for the stability of new and reactivated fear memories.** *Nat Neurosci* 2002, **5**: 348-355.

5. Cardinaux JR., Notis JC, Zhang Q, Vo N, Craig JC, Fass DM, Brennan RG, Goodman RH: **Recruitment of CREB binding protein is sufficient for CREB-mediated gene activation.** *Mol Cell Biol* 2000, **20**: 1546-1552.

6. Kwok RP, Lundblad JR, Chrivia JC, Richards JP, Bachinger HP, Brennan RG, Roberts SG, Green MR, Goodman RH: **Nuclear protein CBP is a coactivator for the transcription factor CREB.** Nature 1994, **370**: 223-226.

7. Brummelkamp TR., Bernards R, Agami R: **Stable suppression of tumorigenicity by virus-mediated RNA interference.** *Science* 2002, **296**: 550-553.
